# Surface orange patinas on the limestone of the Batalha Monastery (Portugal): characterization and decay patterns

**DOI:** 10.1007/s11356-021-15490-1

**Published:** 2021-07-29

**Authors:** Yufan Ding, Pedro Redol, Emma Angelini, José Mirão, Nick Schiavon

**Affiliations:** 1 Hercules Laboratory, University of Évora, Évora, Portugal; 2 Polytechnic of Turin, Turin, Italy; 3 Direção Geral do Património Cultural, Mosteiro da Batalha, Batalha, Portugal; 4 Department of Geosciences, University of Évora, Évora, Portugal

**Keywords:** Oolitic limestone, Orange patina, Scialbatura, Stone decay, Batalha Monastery

## Abstract

Samples of orange patinas found on a limestone window tracery and an ornament of the Batalha Monastery have been investigated by X-ray micro-diffractometry (μ-XRD) and low-vacuum scanning electron microscopy coupled with energy dispersive spectrometry (LV-SEM + EDS). The aim of the study was to determine the composition of the layered patinas, assess whether they have been intentionally applied or naturally formed, and study their degradation patterns. Preliminary results revealed that the orange patinas on the window tracery and the ornament showed different compositions and appearance, suggesting distinct formation pathways. Orange patinas on the ornament, which are now showing decay and delamination patterns, mainly consisted of gypsum with hematite as a minor component, implying the possibility of an intentional application of a mixture of ochre and lime as tint plaster. Orange patinas on the window tracery show, instead, the presence of Ca-oxalates, abundant weddellite, and minor whewellite, with minor hematite suggesting the yellowish/orange color as being due to Ca-oxalate patinas imbedding soil dust airborne particles. Such patina was possibly formed naturally either by the chemical attack due to atmospheric air pollutants from traffic exhausts emissions or by bacterial activity. No delamination was observed on the window tracery sample with granular decohesion as the major decay phenomenon. A comparison was made between this patina and the so-called scialbatura, a surface yellowish coating often found by conservators on limestone and marble in ancient monuments in the Mediterranean region.

## Introduction

**Fig. 1:**
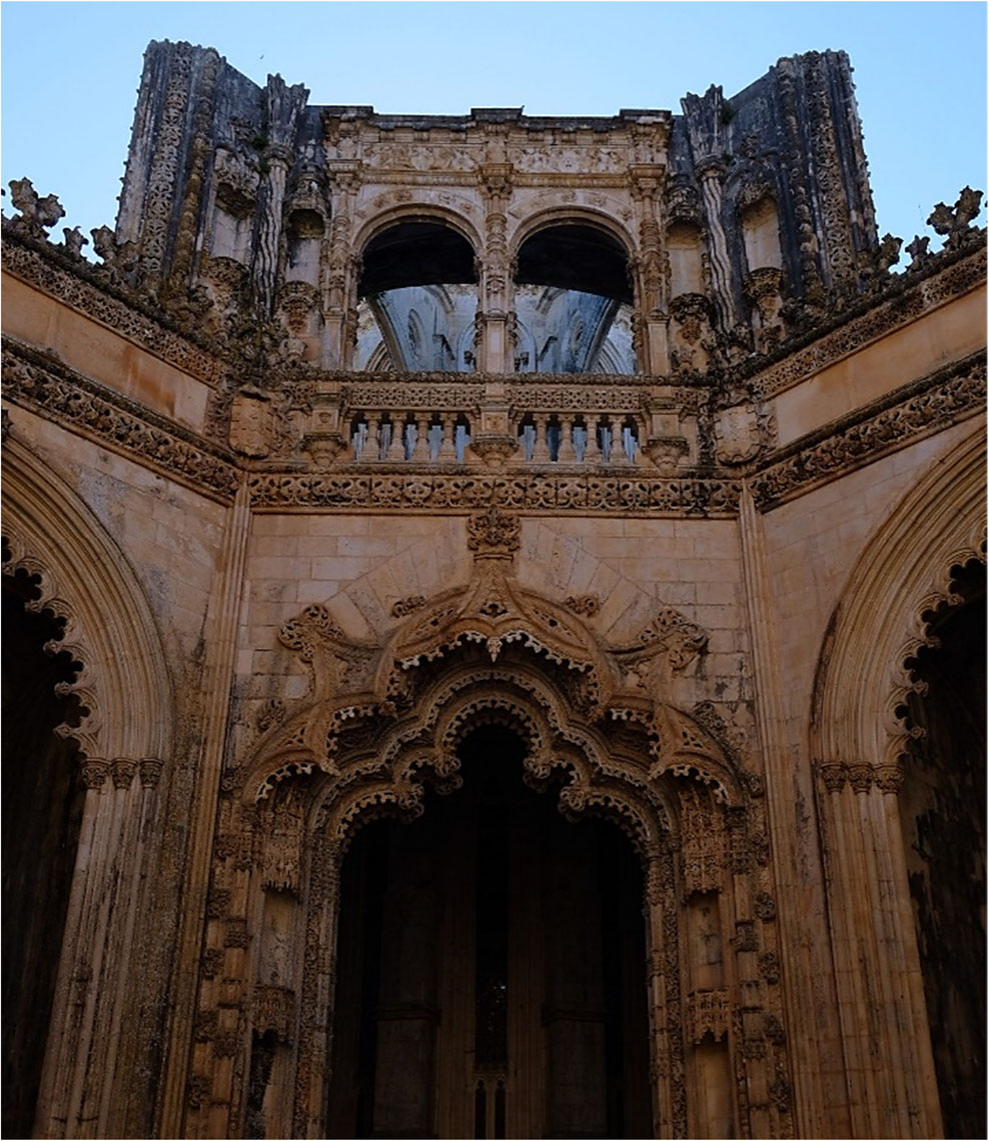
Facade and ornaments with orange patina at the Imperfect Chapels, Batalha Monastery

The famous Batalha Monastery located in the small town of Batalha in Central Portugal was built and restored using Jurassic oolitic limestones extracted from several quarries in the region surrounding the monument (Aires-Barros 2001; Ding et al. 2019). Although originally white in color, the monastery limestone is now extensively covered with orange patinas which can be seen on the surface of facades, ornaments, columns, and walls (Fig. 1). Few studies on this subject can be found in the literature. Rattazzi and coworkers examined two statues stored in the monastery museum: the external orange layers were found to show a rather homogeneous texture with traces of gypsum and clay minerals; FTIR analysis showed bands of Al-silicates and iron oxides, interpreted as Earth of Sienna natural and Red Ochre pigments (Rattazzi et al. 1996). Aires-Barros et al. investigated the original fifteenth-century Saint Matthew statue and the external walls of the main façade. The statue showed the presence of calcite with minor nitrate, silicate, and organic products like benzoic acid, while on the external walls, the orange patinas were found to be predominantly composed by calcium oxalates such as whewellite (CaC_2_O_4_·H_2_O) with minor weddellite (CaC_2_O_4_·2H_2_O), together with hydroxyapatite (Ca_5_(PO_4_)_3_(OH)), halloysite, and nitrates (Aires-Barros et al. 2001).

These results suggested these patinas to represent what conservators called “scialbatura,” e.g., a superficial film present as a surface coating on calcareous Greek and Roman monuments in the urban areas, colored variously yellow, brown, pink, or red and often associated with pitting phenomena (Lazzarini and Salvadori 1989). In the late 1980s, researchers demonstrated that the principal components of the “scialbatura” were Ca-oxalates (mainly weddellite and whewellite), iron oxides (ochres), calcite from the underlying marble substrate, quartz, and feldspars as a result of dry deposition of windborne soil dust, and gypsum originated from the reaction between atmospheric SO_2_ derived from fossil fuel, oil, and coal burning processes and the calcitic substrate (Del Monte and Sabbioni 1987). In fact, the origin of oxalate minerals found on calcitic stone surfaces in urban environments has been the subject of intense debate for decades as being due to different chemical and biogenic processes such as (a) oxalate mineral precipitation associated with chemical attack on calcite-rich substrates of oxalic acid due to metabolic activity of encrusting epilithic lichens (Del Monte et al. 1987; Schiavon 2002; Rosado et al. 2016); (b) non-bio-mediated chemical reactions between the calcareous stone substrate and a number of natural and manmade organic pollutants found in the urban atmosphere from volcanic activity and industrial production activities (Camuffo 1993), and/or vehicle exhaust emissions (Kawamura and Kaplan 1987); and (c) organic compounds used in past decorative or protective treatments and responsible for oxalate formation, such as calcium caseinate, egg, and milk (Lazzarini and Salvadori 1989; Franzini et al. 1984); “Scialbatura” patinas has been also considered to represent intentionally added “sacrificial” coating for the monuments eventually leading, though, to further stone decay (Demitry 1988). In the follow-up studies, Fassina (1995) considered lime as being the main component of the traditional “scialbatura,” with, in addition, titanium oxide detected in the yellow-pink layer. The yellow color was interpreted as related to the presence of fluorite probably formed during the superficial consolidation using fluorosilicate treatments or alternatively to acid cleaning interventions.

Nearly 15 years have passed since the last research carried out on this topic. In this research, two fragments from the Batalha Monastery—an ornament and a window tracery section—have been investigated. Aim of this study was to determine the composition of the orange layers on their surface and make a comparative study between each other and with previous research. Another aim was to assess the possible interactions between the oolitic limestone substrate, the orange surface patinas, and the urban environment surrounding the Monastery.

## Methods and materials

**Fig. 2:**
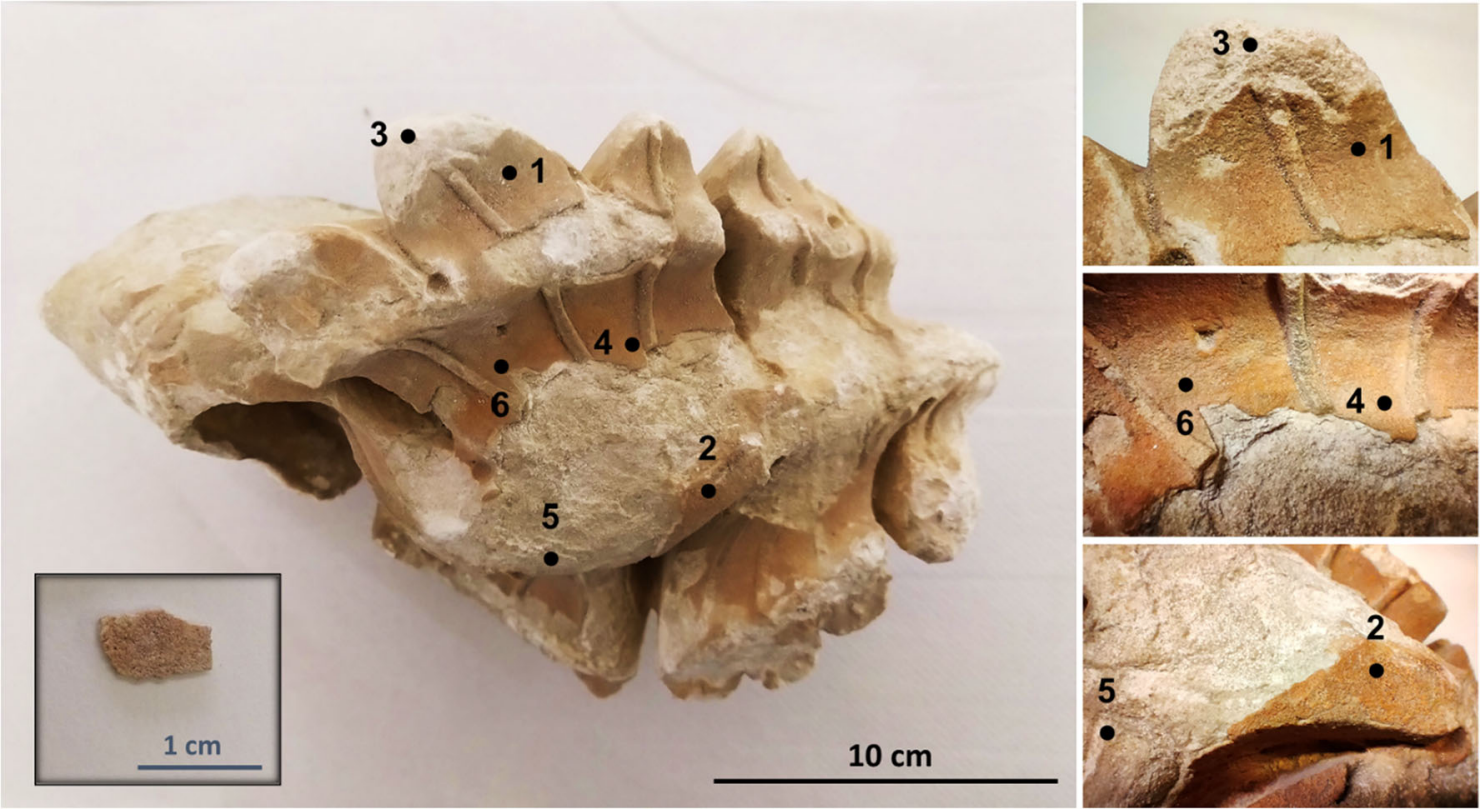
Ornament “S-116” and the peeled-off surface fragment

**Fig. 3:**
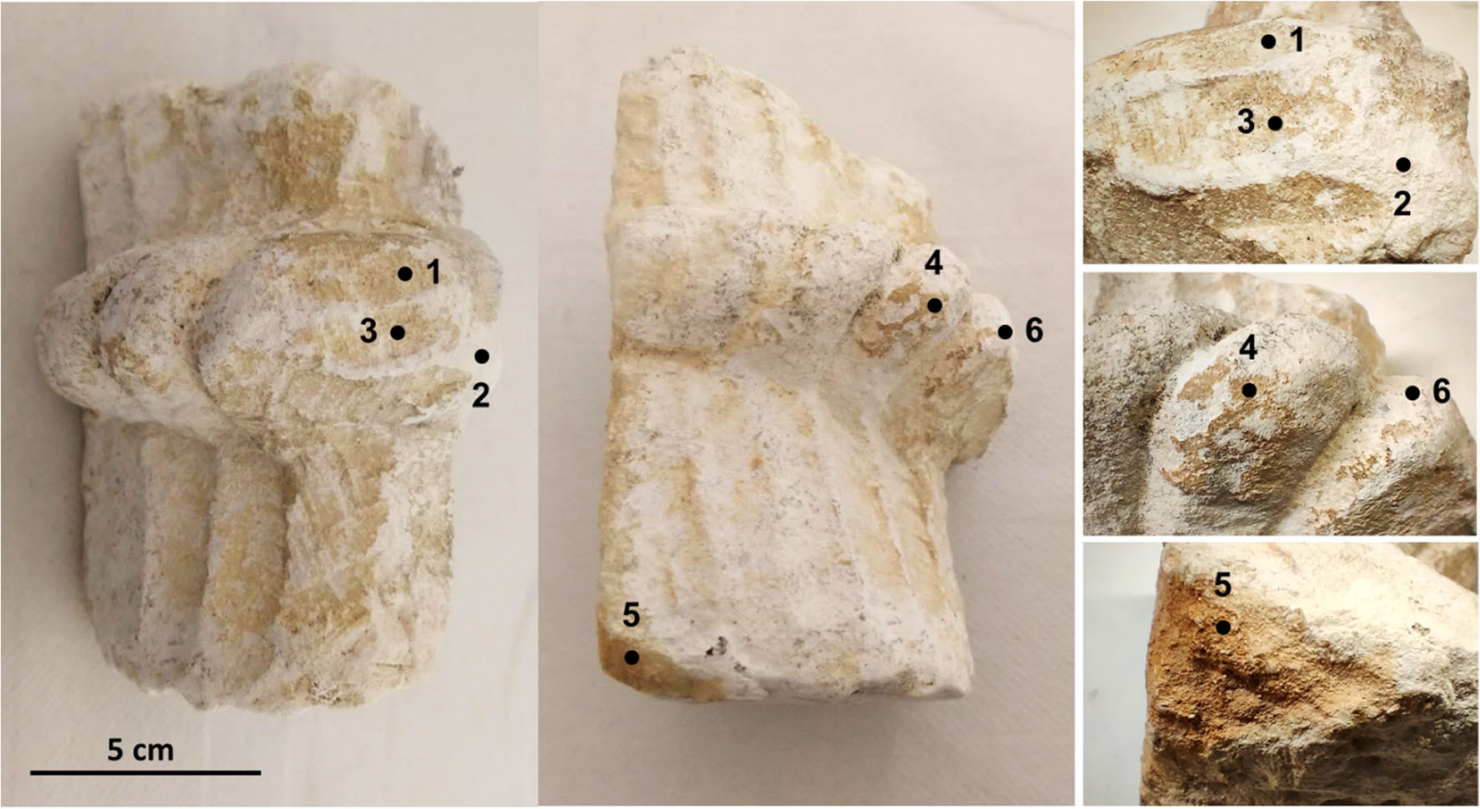
Window tracery piece from the Royal Cloister of the Monastery

By special permission of the Direção-Geral do Patrimonio Cultural and the Mosteiro da Batalha authorities, 2 fragments were sampled from the monastery to HERCULES Laboratory (University of Evora) to carry out the characterization: one is an ornament from the west portal of the church - “S-116” (Fig. 2) from the fifteenth century, and the other is an ornament from a window tracery in the Royal Cloister (Fig. 3). These samples were both located outdoor and occasionally exposed to the sun and rain. Complying with the principle of non-destructive detection, the following analytical methods were used.

### Color evaluation

Datacolor CHECK II PLUS Spectrophotometer was used to determine the color of the patina, by selecting the CIELabCH system; the color evaluation was illustrated as L, a, b c, and H. By comparing the appearance of the samples with the Munsell color chart and taking L, a, and b values as references, color number that is most similar to the presented patinas is selected.

### Optical microscope

Samples were observed under LEICA M205C Stereo Microscope (Leica, Wetzlar, Germany). A peeled-off fragment of sample S-116 was consolidated with resin and polished, for the observation of the cross-section.

### X-ray micro-diffractometry (μ-XRD)

Multiple points on the orange layer and the substrate of the samples (Fig. 2 and Fig. 3) were characterized by μ-X-ray diffraction using a commercial Bruker AXS D8 Discovery diffractometer with Cu Ka radiation, Lynxeye detector, interval 3–70° 2μ, and step of 0.028/s. DIFFRAC.SUITE EVA and Highscore Plus software were used to identify the mineralogical composition and to perform a semi-quantification by the RIR method (Hubbard and Snyder 1988).

### Variable-pressure scanning electron microscopy coupled with energy dispersive spectrometry (VP-SEM + EDS).

Scanning electron microscopy coupled with energy dispersive X-ray spectrometry was carried out using a Hitachi S3700N (Tokyo, Japan) SEM coupled to a Bruker (Karlsruhe, Germany) XFlash 5010 SDD Detector system (Veiga et al. 2014). The samples were characterized at a chamber pressure of 40 Pa, without any sample preparation, with an accelerating voltage of 20 kV, using a working distance 10 mm and a BSEM detector.

## Results and discussion

**Fig. 4:**
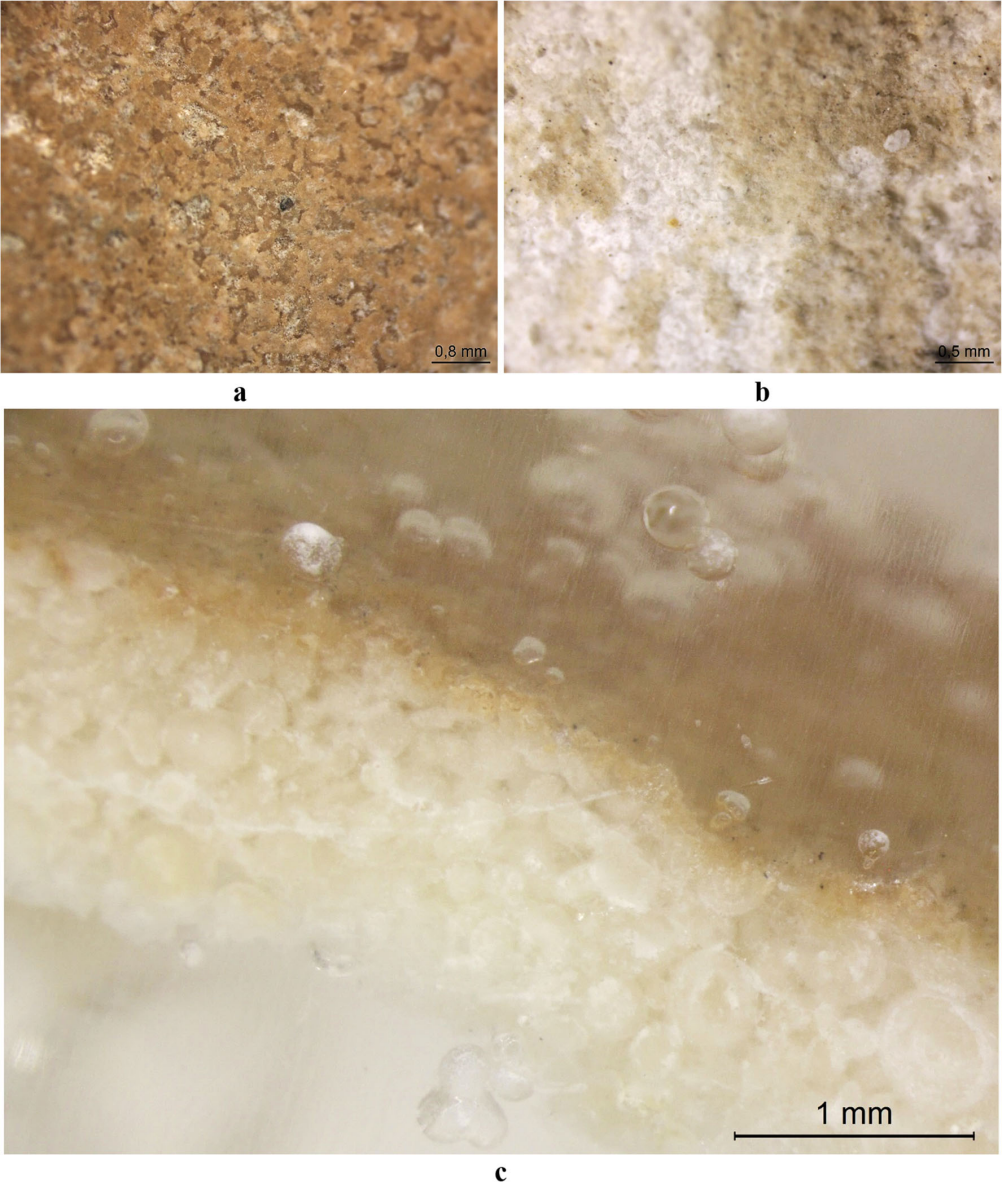
Optical microscope photos of **a** sample S-116 surface, **b** the window tracery sample surface, and **c** cross-section of the peeled-off fragment from sample S-116

The photographs of the sample S-116 and window tracery piece are presented in Fig. 2 and Fig. 3, respectively. Microscopy was performed on these two samples, and the pictures are shown in Fig. 4. It can be seen that the orange color of the patina on S-116 is more saturated than that of window tracery sample. The patina on the window tracery surface is unevenly distributed, and its color also shows variegation over the area (Fig. 4b). On the contrary, the patina on the ornament S-116 is much more homogenous, and the color is consistent over the patina covered region (Fig. 4a). Cross-section observation revealed that the peeled-off fragment consists of a 0.1~0.2 mm orange layer and 1~1.2 mm oolitic limestone substrate, and the fragment has thickness of ~1.3 mm (Fig. 4c), implying that the detachment of this coating happens under the limestone surface.

### Ornament “S-116” from Batalha Monastery

The color evaluation of the patina on sample S-116 is L: 68.63, a: 9.66, b: 15.67, C: 16.56, and H: 66.04. MUNSELL 2.5YR/6/6 is very close to this color.

**Fig. 5:**
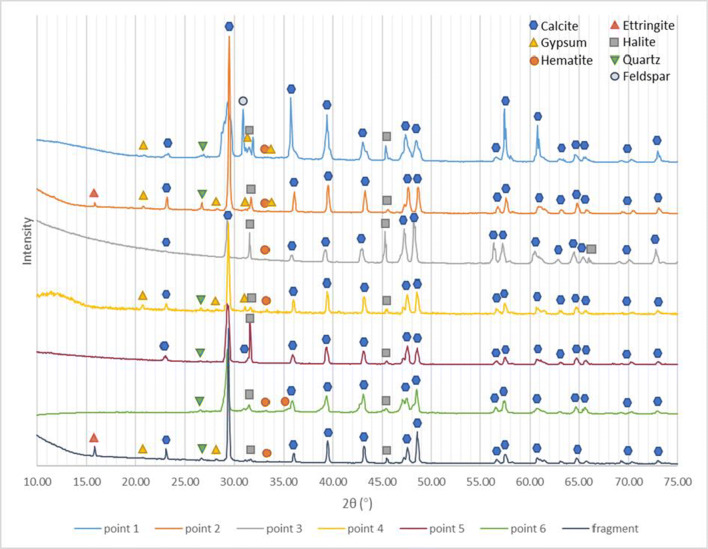
XRD results of multiple points on ornament “S-116” and the peeled-off fragment

μ-X-ray diffraction patterns were acquired on six points of the ornament “S-116” and a peeled-off fragment, as shown in Fig. 2. Points 1, 2, 4, and 6 were acquired on the orange surface patinas, whereas points 3 and 5 were acquired on the substrate where the orange surface had already peeled off with the limestone substrate showing extensive decay. The XRD pattern and peaks are presented in Fig. 5, and a semi-quantitative evaluation is given in Table 1.

**Table. 1:** Qualitative and semi-quantitative list of minerals and compounds detected on the ornament “S-116” surface

Composition	Fragment	Point 1	Point 2	Point 3	Point 4	Point 5	Point 6
	Appearance						
	Orange surface	Orange surface with dust	Orange surface	Weathered white substrate	Orange surface	Weathered white substrate	Orange surface
Calcite (CaCO_3_)	81%	40%	74%	70%	73%	72%	68%
Gypsum (CaSO_4_•2H_2_O)	7%	20%	8%	-	19%	-	9%
Quartz (SiO_2_)	2%	4%	5%	7%	3%	7%	6%
Hematite (Fe_2_O_3_)	1%	5%	2%	2%	3%	-	14%
Halite (NaCl)	1%	5%	4%	21%	2%	21%	3%
Ettringite	8%	-	7%	-	-	-	-
Feldspar (KAlSi_3_O_8_)	-	26%	-	-	-	-	-

In both samples, the stone substrate is an oolitic limestone consisting mainly of calcite and quartz (Ding et al. 2019): accordingly, peaks of both minerals are seen at all points analyzed. A considerable amount of gypsum can be detected at points 1, 2, 4, and 6 and in the detached fragment, in areas corresponding to the orange surface, while at points 3 and 5 where the coating is completely removed, gypsum is absent. Halite was present in all analyzed spots, with its content being significantly higher at points 3 and 5, indicating that halite was concentrated on the substrate surface under the orange layer. Feldspar peaks (Al-K-silica) were present particularly at point 1, confirming the presence of soil dust deposition in this area. A minor amount of hematite was detected in all orange patinas suggesting the presence of red ochre pigment.

The sporadic presence of ettringite peaks in XRD results is somewhat intriguing. The presence of ettringite could be due to the cementitious materials exposed to sulfate attack: calcium aluminate hydrates and calcium silicate hydrates mixed with lime can react with water and gypsum or other sulfate salts, to produce ettringite [Ca_6_Al_2_(SO_4_)_3_(OH)_12_·26H_2_O] and thaumasite [Ca_3_Si(OH)_6_(CO_3_)(SO_4_) 12H_2_O] (Collepardi 1999). The application of cement-based mortar in the Batalha monastery during past restoration interventions (Soares 2001) could therefore explain the presence of ettringite on the ornament surface.

**Fig. 6:**
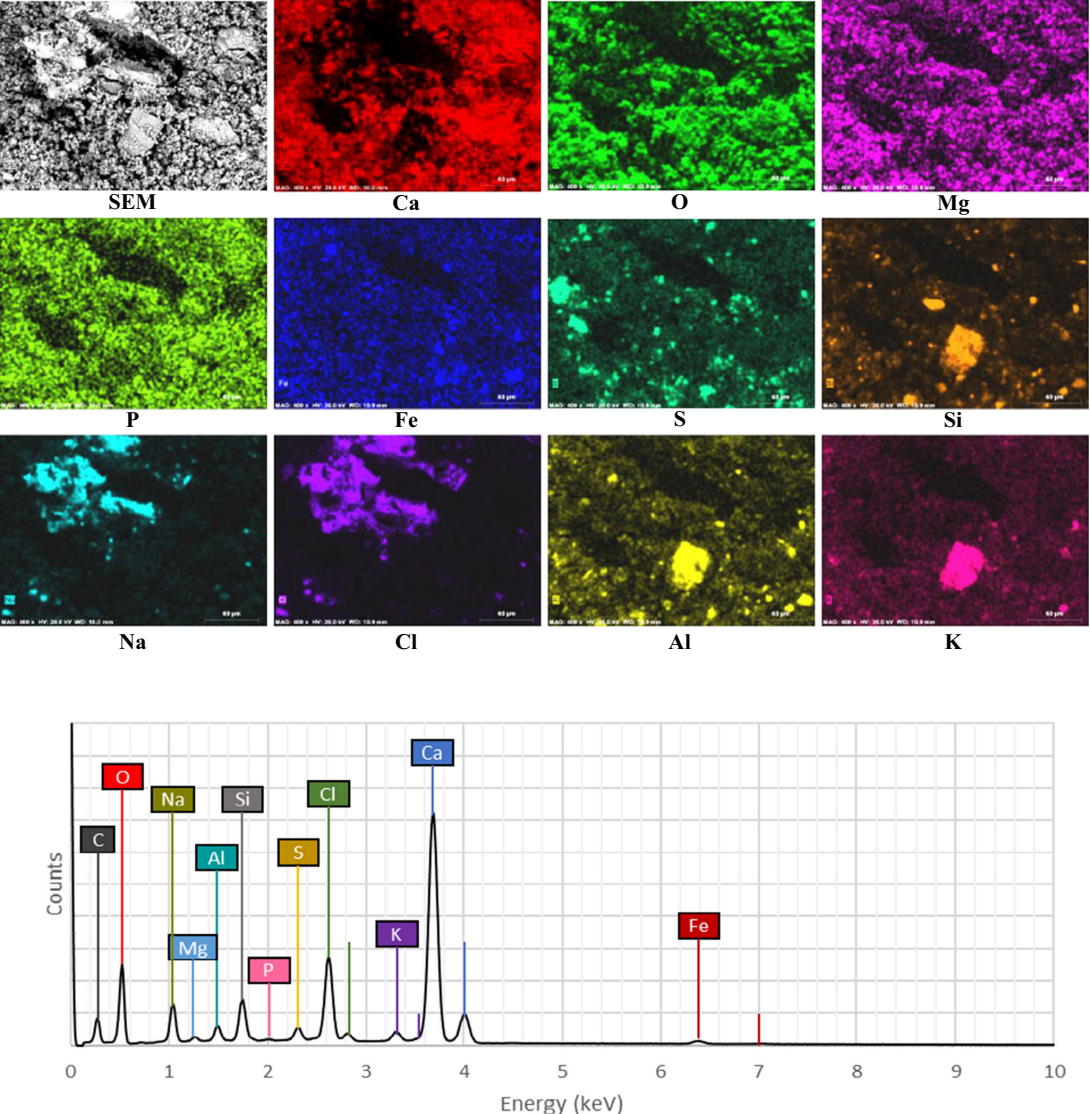
SEM-EDS element mapping of ornament “S-116” fragment

The peeled-off fragment was observed under the VP-SEM+EDS: results are shown in Fig. 6. There is a correlation between the distribution of sodium and chloride, demonstrating the presence of halite (NaCl) grains. Potassium (K), aluminum (Al), and silicon (Si) peaks imply the presence of quartz, feldspar, and clay minerals probably as soil dust. The peak of calcium (Ca) is weak where the NaCl and feldspar/soil dust/clay grains are present, which means halite and aluminosilicates were formed subsequently on the top of the limestone substrate. This could also be seen from the SEM picture; the halite (up left) and feldspar (down central) show a well-formed crystalline habit with relatively large dimension grains (>50 μm), in contrast with the poorly crystallized gypsum grains.

**Fig. 7:**
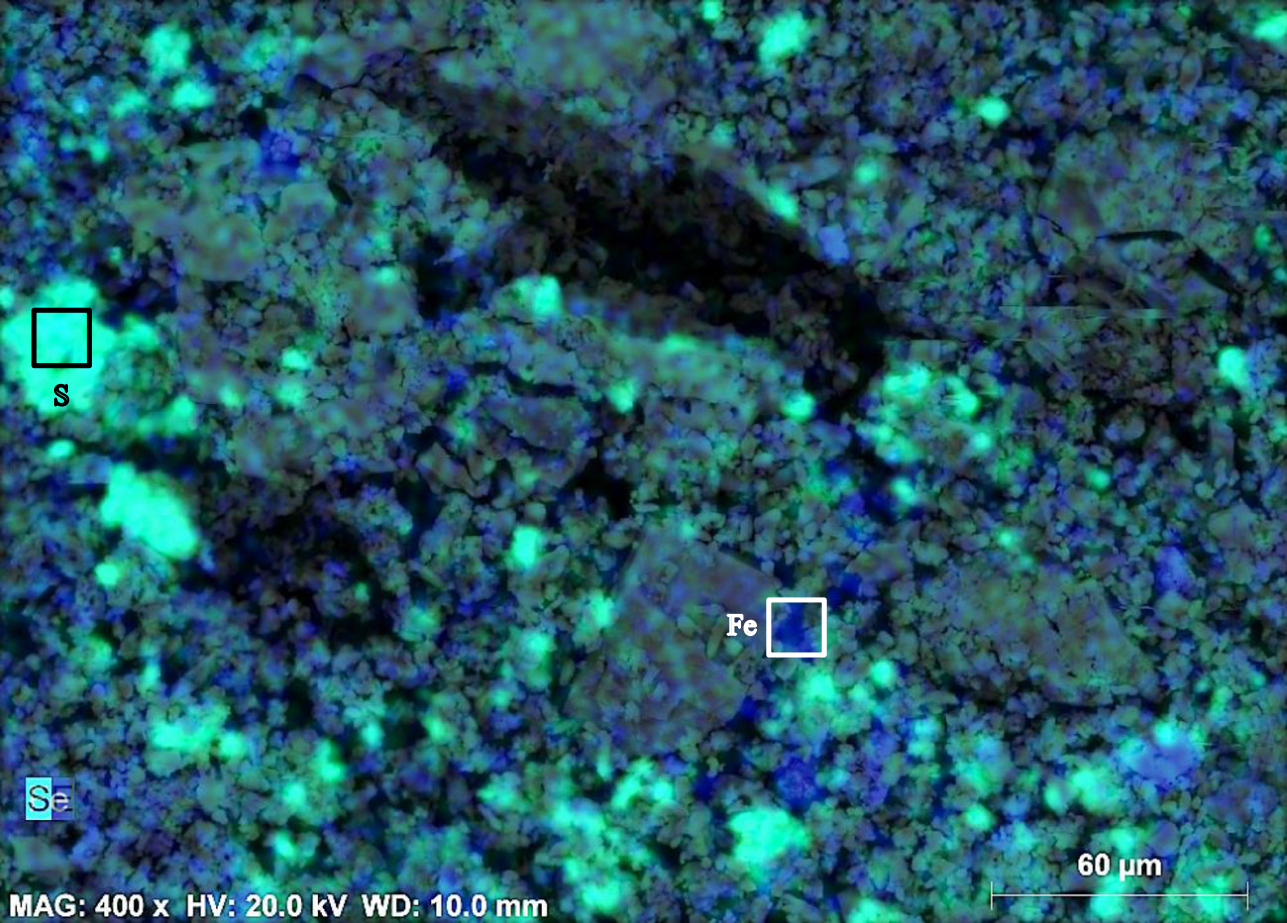
Overlapped element mapping of Fe (blue) and S (neon green) with the morphology photo

The distribution of iron (Fe) was homogenous over the whole analyzed area, supporting the interpretation of a pigment surface application. On the other hand, hematite is also present as accessory mineral grains in the original limestone. The presence of sulfur (S) is mainly due to gypsum (CaSO_4_•2H_2_O); its distribution is uniform, and some multi-mineral grains can be seen. By overlapping the distribution of Fe and S (Fig. 7), it can be seen that hematite was likely to be mixed and applied together with gypsum on the uppermost layer. This hypothesis can be supported by the recorded use of red and yellow ochre: in ancient Macedonian paintings, one of the main means of using ochre was to mix them with lime to produce tint plasters which were then applied at the surface of monuments (Perdikatsis and Brecoulaki 2008). Gypsum plaster might have been applied in the lime to achieve faster setting time. Therefore, the color on the ornament “S-116” surface could be the reason of the same procedure. It is noteworthy the presence of phosphorous (P), which was also detected by Lazzarini and Salvadori (1989) on the “scialbatura” of Cathedral and church of S. Zeno, Verona, and interpreted as an artificial protective coating made from calcium caseinate.

### Window tracery from Batalha Monastery Royal Cloister

The color evaluation of the patina on the cloister window tracery is L: 85.24, a: 3.97, b: 15.46, C: 15.96, and H: 75.58. MUNSELL 5YR/8/6 is very close to this color.

The window tracery sample was analyzed only by micro-XRD to prevent causing any damage. XRD results of multiple points on the Royal Cloister window tracery shows the XRD pattern, and in Table 2, the main minerals are listed together with their semi-quantitative evaluation. At point 1, where an orange surface is still visible, abundant calcium oxalate was present including weddellite and whewellite, together with feldspar and hematite. Gypsum was detected where the orange surface was worn-out and detached revealing the underlying white substrate. In these areas, the main composition was calcite with minor amount of gypsum and calcium oxalate and no detected hematite. Point 3 has a different appearance than point 1, the surface showing a dark orange color, and the texture being loose and powdery in this area; the XRD results revealed calcite, some weddellite, feldspar, and minor hematite. Halite was present at point 3 and absent at points 1 and 2.

**Table. 2:** Qualitative and semi-quantitative list of minerals and compounds detected on the window tracery

Composition	Point 1	Point 2	Point 3	Point 4	Point 5	Point 6
	Appearance					
	Orange surface	White substrate	Orange surface	Orange surface	Orange surface loose texture	White substrate
Calcite (CaCO_3_)	54%	80%	15%	38%	85%	100%
Gypsum (CaSO_4_•2H_2_O)	18%	9%	39%	19%	3%	-
Weddellite (CaC_2_O_4_·2H_2_O)	17%	10%	31%	37%	7%	-
Whewellite (CaC_2_O_4_·H_2_O)	9%	-	12%	-	-	-
Quartz (SiO_2_)	2%	1%	3%	6%	2%	-
Hematite (Fe_2_O_3_)	-	-	-	-	1%	-
Halite (NaCl)	-	-	-	-	2%	-

**Fig. 8:**
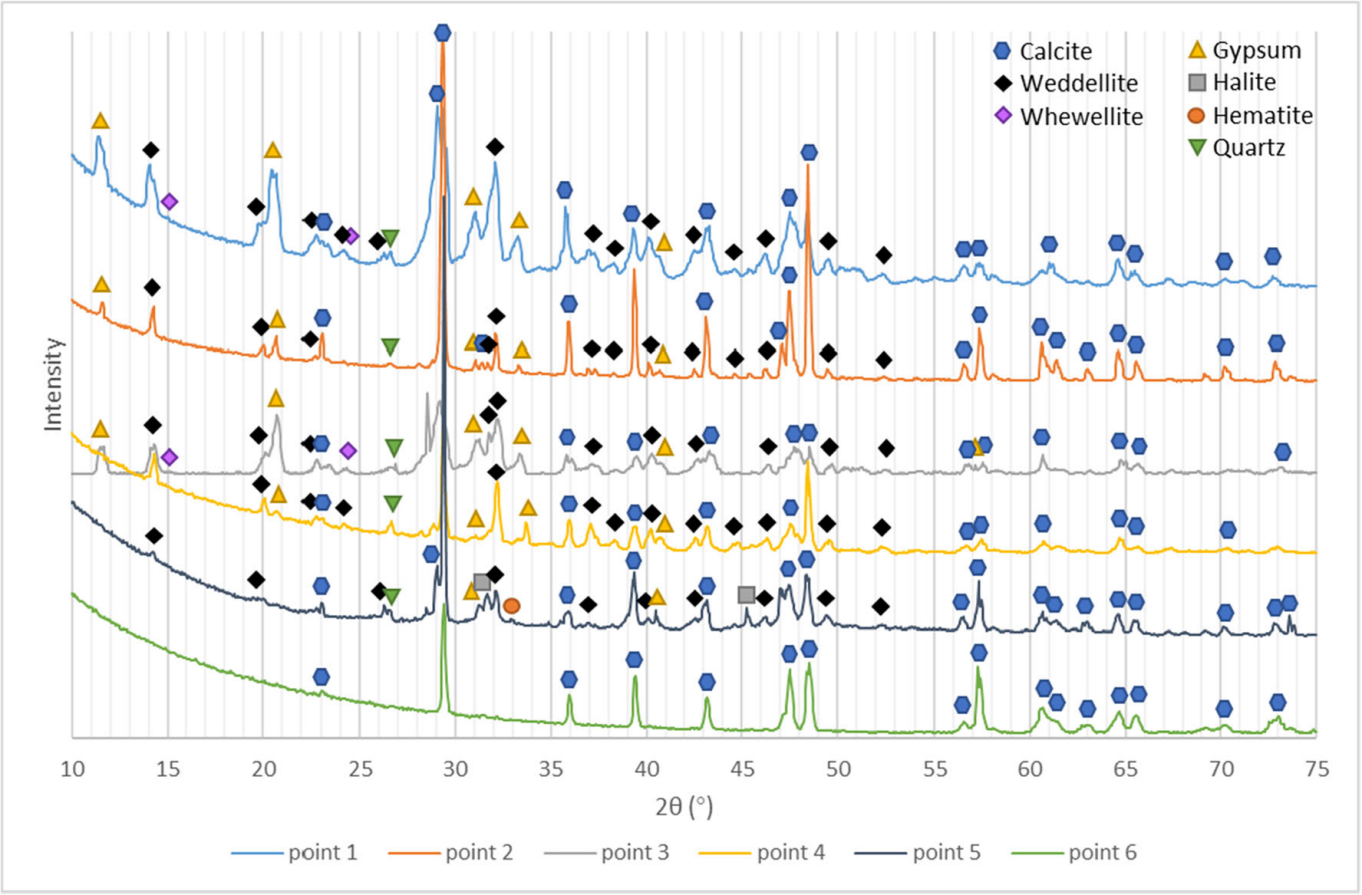
XRD results of multiple points on the Royal Cloister window tracery

Calcium oxalate is itself colorless, but when organic compounds, mainly derived from the metabolic activity of lichens, fungi, and bacteria (from oxalic acid which reacts with calcite to form a thin calcium oxalate film) and other mineral grains (such as quartz and feldspar) are present, the patina may acquire a yellowish-brown hue (Ion et al. 2017). Thus, the color on this window tracery could possibly originate from calcium oxalate mixed with other mineral grains such as soil dust. The stone surface oxalates were generally considered to be the result of lichen excreted oxalic acid that reacts with calcium in the substrate (Del Monte et al. 1987); however, a few recent studies reported that various bacteria can produce oxalic acid even without lichen microbiomes, for instance, *Pseudomonas fluorescens*, *Burkholderia*, *Bacillus*, and *C. jiangningensis JN53* (Palmieri et al. 2019; Hess et al. 2008; Cheng et al. 2017). In fact, *Bacillus* (phylum Firmicutes) and *Burkholderia* (phylum Beta-Proteobacteria) were found in the Batalha Monastery bio-deteriorated stone and the atmospheric environment in a previous study by the same authors (Ding et al. 2021). Thus, although no lichen crust was present on this Window tracery sample, the possibility of bacteria producing oxalic acid and causing mineral weathering cannot be excluded (Fig. 8).

Calcium oxalates may also be derived from vehicular exhaust emissions with incomplete combustion of aromatic hydrocarbons (such as benzene, toluene, and naphthalene) in the car engines generating diacids including oxalic acid, cis-unsaturated acids, and aromatic acids (Kawamura and Kaplan 1987). Due to the polar nature of these dicarboxylic acids, they preferentially associate with moisture and could react with calcareous stone and form calcium oxalates. As a matter of fact, the Portuguese national highway IC2 run through Batalha only 50 m west of the monastery, so atmospheric vehicular pollution could not be ruled out as a cause for the development of oxalate patinas on the limestone surfaces of the monastery. The benzoic acid found by Aires-Barros et al. (2001) on the external walls of this monument makes this hypothesis more plausible.

### Comparison between the two samples

Though both named “orange patinas,” there are significant differences between the two samples investigated. Table 3 lists these differences with respect to composition, appearance, and decay patterns. For the ornament “S-116,” the orange layer follows the external geometrical shape of the object, for example, the ridging stripes spreading out from the center, which also existed on the substrate. For the Royal Cloister window tracery, the orange patina is closely adherent to the limestone substrate instead of a visible separate layering present on “S-116.” And there was an abundant weddellite on the window tracery surface, which was not found on the sample “S-116.”

**Table. 3:**
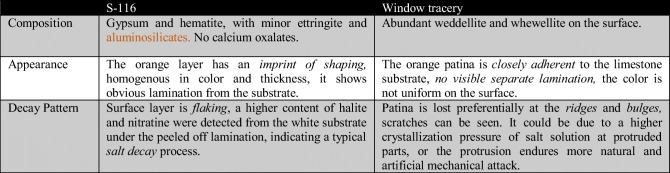
Comparison between the orange patinas on the sample “S-116” and on the Royal Cloister Window tracery.

The decay pattern of these orange layers on the two samples was also distinct. On the ornament “S-116,” the surface layer was flaking; on the white substrate under the peeled-off lamination, higher content of halite was detected. This indicates a typical salt decay process: salt solution evaporated and crystallized at the interface between the coating and the substrate, leading to detachment eventually (Duffy and Perry 1996). In this case, the salt responsible for decay is mainly halite (NaCl); its precipitation leading to salt decay in the monastery may probably derive from ground-moisture capillary rise. Considering the Royal Cloister window tracery, the patina was lost preferentially at the ridges and bulges but was more preserved in flat areas where marks of scratches can also be seen. Three possible explanations can be suggested: (a) the evaporation rate of salt solutions is higher at the geometrically protruded parts due to a larger specific surface area, resulting in higher crystallization pressure inside the stone pores and more severe decay (Rodriguez-Navarro and Doehne 1999); (b) protruded areas endure more mechanical abrasion from rain, wind, sands, and human activities, thus accelerating the decay processes; or (c) the orange patina was originally formed irregularly on the substrate.

**Fig. 9:**
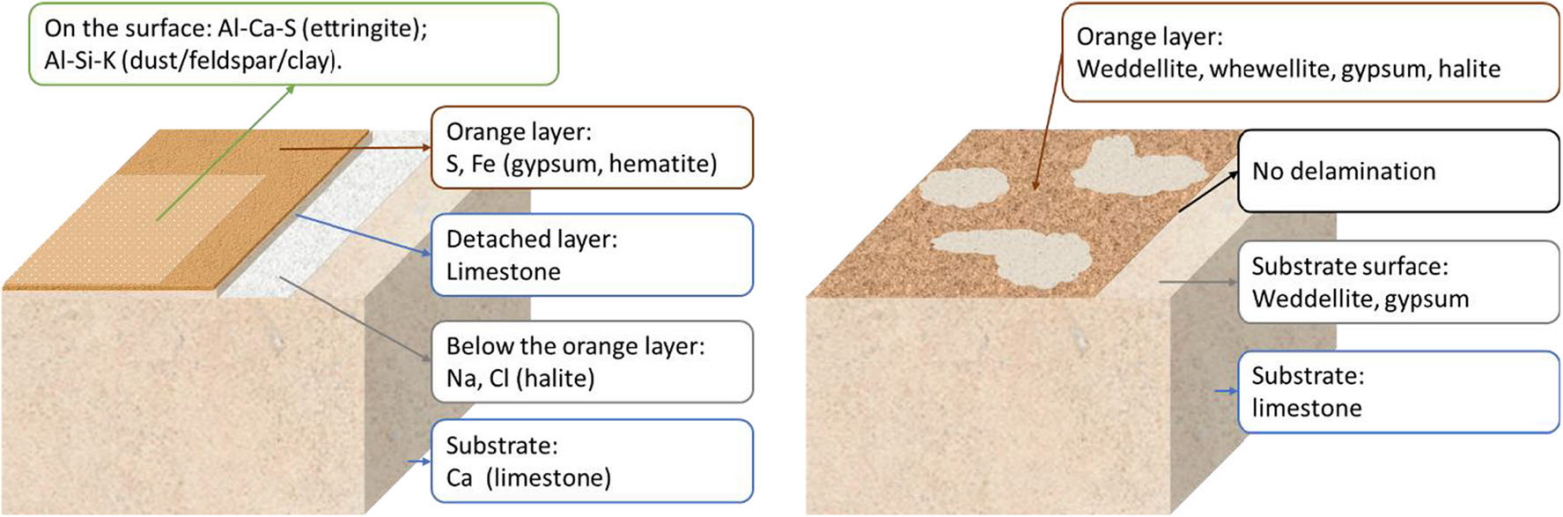
Sketch of the sample “S-116” (left) and the Window tracery (right)

Combining the results obtained, preliminary sketch (Fig. 9) was made to elucidate the different characteristics of the two samples investigated.

### Comparison with the previous research

Through the above-mentioned results and analysis, it can be concluded that the orange layer on the Royal Cloister window tracery is similar to the findings by Aires-Barros et al. (2001). Such patina is compatible with a “scialbatura” nature for its composition, texture, and color, as described by Lazzarini and Salvadori (1989). No titanium oxide and fluorite presence as reported in previous research (Fassina 1995). The results of the present study are in line with the conclusion that the presence of calcium oxalates on this monastery could be linked to either vehicular exhaust emissions (Kawamura and Kaplan 1987) or to bacterial activity (Palmieri et al. 2019), but not to oxalic production by lichenous colonization and its reaction with the calcite-rich stone surface (Del Monte and Sabbioni 1987; Schiavon 2002).

The orange coating on the ornament “S-116” sample matches the description of that on the original apostle statues from the church doorway (Rattazzi et al. 1996). Considering the ornament “S-116” and the apostle statues were both crafted in the fifteenth century, it is feasible to consider that the same coating procedure was used, despite them being located in different sites of the Batalha Monastery. Although it is commonly believed that in this type of surface layers, the gypsum occurring on calcareous stones is a reaction product of calcareous or silicate stones in a SO_2_-polluted urban atmosphere (Schiavon 2007), the possibility of artificial gypsum coating cannot be completely excluded (Sánchez et al. 2009). In fact, the homogeneity of the layers and the presence of hematite may support the conclusion of the intentional application of a pigmented plaster.

## Conclusion

In this research, a chemical/mineralogical characterization was carried out on orange surface patinas found in two different limestone artifacts from Batalha Monastery in Portugal. The homogenous and decorative layer on ornament “S-116” is mainly composed of gypsum and hematite with traces of K-feldspars possibly from soil dust and mortar joints in the monument. It is highly likely to be applied intentionally, using the same craft procedure as the apostle statues of the monastery. Its delamination is a typical phenomenon of salt decay, for high concentration of halite was found on the substrate under the exfoliated surface.

The colored surface of the Royal Cloister Window tracery contains abundant calcium oxalate including weddellite and whewellite; gypsum was also detected. The surface color was not homogeneous on the window tracery piece where erosive episodes led to surface loss. Based on the above results, this surface layer was compatible with the description of “scialbatura,” as it shows evidence of substrate stone reacting with the environment. This patina was likely generated due to the air pollution and the bacterial metabolic activities.

## Data Availability

Data is contained within the article.
